# RNA-Binding Proteins: Emerging Therapeutics for Vascular Dysfunction

**DOI:** 10.3390/cells11162494

**Published:** 2022-08-11

**Authors:** Victoria A. Cornelius, Hojjat Naderi-Meshkin, Sophia Kelaini, Andriana Margariti

**Affiliations:** Wellcome-Wolfson Institute for Experimental Medicine, School of Medicine, Dentistry and Biomedical Sciences, Queen’s University Belfast, 97 Lisburn Road, Belfast BT9 7BL, UK

**Keywords:** vascular disease, RNA-binding proteins, stem cell technologies, iPSCs, Quaking, QKI

## Abstract

Vascular diseases account for a significant number of deaths worldwide, with cardiovascular diseases remaining the leading cause of mortality. This ongoing, ever-increasing burden has made the need for an effective treatment strategy a global priority. Recent advances in regenerative medicine, largely the derivation and use of induced pluripotent stem cell (iPSC) technologies as disease models, have provided powerful tools to study the different cell types that comprise the vascular system, allowing for a greater understanding of the molecular mechanisms behind vascular health. iPSC disease models consequently offer an exciting strategy to deepen our understanding of disease as well as develop new therapeutic avenues with clinical translation. Both transcriptional and post-transcriptional mechanisms are widely accepted to have fundamental roles in orchestrating responses to vascular damage. Recently, iPSC technologies have increased our understanding of RNA-binding proteins (RBPs) in controlling gene expression and cellular functions, providing an insight into the onset and progression of vascular dysfunction. Revelations of such roles within vascular disease states have therefore allowed for a greater clarification of disease mechanisms, aiding the development of novel therapeutic interventions. Here, we discuss newly discovered roles of RBPs within the cardio-vasculature aided by iPSC technologies, as well as examine their therapeutic potential, with a particular focus on the Quaking family of isoforms.

## 1. Introduction

Vascular diseases encompass a range of pathological conditions that affect blood vessels, many of which can lead to the onset of a range of fatal complications. Accordingly, cardiovascular diseases (CVDs) are one of the leading causes of death worldwide [1]. Although vascular diseases can occur across the circulatory system and because of numerous factors, all occur through the onset of endothelial cell (EC) dysfunction. ECs form a vast and complex monolayer across the innermost lining of all blood vessels, known as the endothelium [2]. The extraordinary plasticity of the endothelium allows for it to respond to a variety of stimuli, and as such, ECs are instrumental regulators of vascular homeostasis [3]. This is further illustrated by ECs’ ability to regulate smooth muscle cell (SMC) proliferation as well as control vascular tone and cell adhesion in a precise manner via the secretion of vasoprotective factors. Similarly, vascular SMCs have many critical roles in maintaining the appropriate physiological functions of blood vessels, including regulating vasoconstriction, vasodilation and extracellular matrix production. Likewise, their dysfunction also contributes to the pathogenesis of various vascular diseases. Research has shown the impaired function of both ECs and vascular SMCs to result in serious vascular complications, such as increased permeability, disturbed vascular tone, aberrant angiogenesis, enhanced adhesion and monocyte and platelet deposition. This dysfunction consequently leads to blood vessel damage through thrombogenesis and thus contributes to the onset of vascular diseases. The correct functioning of both ECs and vascular SMCs is therefore essential for a healthy endothelium and, subsequently, vascular health.

Although there are various treatment options that have led to a reduction in general mortality rates, there are currently no gene therapies or efficacious drugs able to restore blood vessel damage. The repair and regeneration of healthy blood vessels is consequently an important focus in regenerative medicine to improve vascular health [4]. Sampling both ECs and vascular SMCs from patients would allow for the identification of potential therapeutic strategies; however, currently, there are no effective methods to directly obtain these cell types. Stem cell technologies are therefore further accelerating the advancement of regenerative medicine by providing patient-derived disease models of vascular cells [5,6]. Due to the differentiation capabilities of stem cells, in particular, of induced pluripotent stem cells (iPSCs), reprogramming technologies have offered strategies to generate patient-specific disease models with evidence that these model cells have therapeutic efficacy in animal models of disease [7,8]. The unique characteristics of iPSCs have allowed for the development of protocols to effectively reprogram iPSCs into both functional ECs and vascular SMCs. The development of such monolayers has aided our understanding of specific roles of certain cell types within the vasculature. It is also important, however, to consider the effect of cell–cell interactions and cell–ECM interactions within health and disease states, too. As such, studies have repeatedly emerged using coculture techniques combining iPSC-derived cell types to generate vascular networks for their investigation [9,10,11,12]. More recently, human blood vessel organoids containing iPSC-derived vascular cell types self-assembled into capillary networks enveloped by a basement membrane have acquired great interest due to their ability to recapitulate the structure and function of human blood vessels. Consequently, both 2D to 3D patient-derived iPSC models can provide functional vascular systems allowing for the study of disease pathophysiology and therapeutics that will have a direct clinical relevance.

Such models have demonstrated the ability of RNA-binding proteins (RBPs) to exhibit control over cellular phenotypes, as well as play a vital role in health and disease states. RBPs are proteins with the ability to bind to RNA and form ribonucleoproteins, which are then subsequently involved in a wide range of processes, including alternative splicing, localisation, stability and translation. As a result, RBPs can significantly alter RNA fate through a number of post-transcriptional mechanisms [13]. In recent years, RBPs have emerged as critical regulators of the vascular system, with roles ranging in development and function to stress management and disease formation. Whilst this review focuses on the blood vascular system, it is also noteworthy to mention a role of RBPs within the lymphatic vascular system. RBPs have emerged as regulators within physiological processes, such as lymphangiogenesis, as well as disease progression, including cancer development due to the role of the lymphatic vessels in the dissemination of tumour cells, both of which have been reviewed extensively elsewhere [14,15]. Regardless of vascular system, exploration of RBP involvement in health and disease states has emerged as a desirable area of research allowing for a greater understanding of cellular physiology and disease pathology [16].

Remarkably, research has revealed that reversing the effects of dysregulated RBPs can be beneficial in alleviating their associated complications, highlighting the potential of RBPs to become effective therapeutic strategies. The RBP Human Antigen R (HuR), for instance, has previously and repeatedly been implicated in the development of vascular diseases, with research displaying HuR expression to positively correlate with the presence and severity of cardiac and vascular dysfunction [17]. Typically, HuR has numerous beneficial physiological roles within ECs, such as regulating inflammatory responses, stabilising angiogenic factors and promoting cellular proliferation and migration [18], whilst in vascular SMCs, HuR regulates contractions and, subsequently, blood pressure [19]. Dysregulation of HuR, however, is heavily involved in the development of cardiovascular complications associated with diabetes, including diabetic retinopathy [20] and nephropathy [21,22]. However, restoration of HuR function both stabilised and ameliorated the associated observed vascular defects, indicating that HuR may be an effective therapeutic target to alleviate certain vascular complications [23,24]. Accordingly, there is a current, growing consensus that RBPs have great potential to become beneficial therapeutic strategies to tackle various vascular disorders. More recently, the Quaking (QKI) RBPs have been of particular interest due to recent research unveiling their extensive involvement within the cardiovascular network, including disease progression.

In this review, we explore the roles of RBPs within the vascular system with emphasis on their therapeutic potential. In particular, we review the recent findings aided by stem cell technologies that highlight the involvement of the QKI RBP isoforms within vascular health.

## 2. RBPs Are Fundamental Regulators of RNA Fate

**Figure 1:**
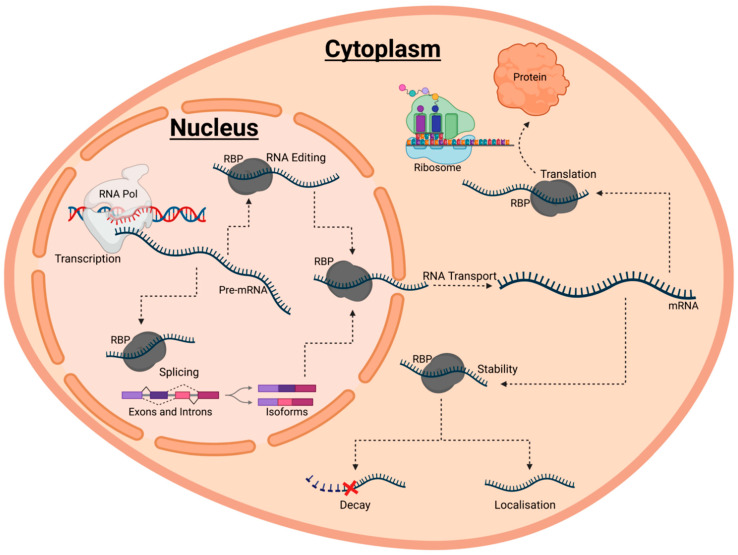
A schematic diagram summarising the various roles of RNA-binding proteins (RBPs) in post-transcriptional gene regulation (PTGR). PTGR encompasses the maturation, transport, stabilit, and translation of coding and non-coding RNAs. Each of these events are heavily regulated by ribonucleoprotein complexes with RBPs at their core. Typically, RBPs are first involved in the maturation of RNA through regulating processes such as RNA editing and splicing. Following this, mature RNA transcripts are exported to the cytoplasm by various RBPs. Within the cytoplasm, RBPs then coordinate the localisation of RNAs to subcellular compartments, as well as orchestrate either the degradation or stabilisation of RNA by binding the substrate RNAs. Stabilisation results in the translation and folding of RNAs into the corresponding proteins constructs.

The human genome codes for a total of ~20,000 genes. To produce the spectrum of proteins responsible for the higher degree of complexity observed in humans, 95% of these coded genes will undergo post-transcriptional gene regulation (PTGR). Moreover, recent evidence suggests that RNA level alone is not a correct index of determining gene expression level but post-transcriptional RNA modifications, so-called the epitranscriptomic code or epitranscriptome, determine the actual gene expression profile at protein level [25]. PTGR encompasses the maturation, transport, stability and translation of RNAs and is primarily orchestrated through the multi-functional roles of RBPs, many of which act in concert [26,27]. It is predicted that the human genome encodes at least 1,542 RBPs [28]. Furthermore, research has revealed RBPs to be amongst the most abundant proteins in cells and in general are ubiquitously expressed, emphasising their centrality within gene regulation. RBPs have established RNA-binding domains that are able to target various types of RNA including single stranded, double stranded, stem loop and tertiary folded due to containing RNA recognition motifs (RRMs), heterogenous ribonucleoproteins (hnRNP) K-homology domains (KH) and C3H1 zinc-fingers (ZF) [29]. A schematic overview of the primary roles of RBPs within PTGR can be seen in Figure 1. Briefly, however, through direct binding to mRNAs, RBPs control pre-mRNA splicing [19], mRNA stability [30] and mRNA translation efficiency [16,26] and thus alter RNA fate in a cell dependent manner to result in a tissue-specific protein repertoire and cellular function [31,32,33]. As we previously discussed, at least 74% of human genes, through the actions of RBPs, are alternatively spliced resulting in the production of multiple isoforms as later observed with the QKI family [34]. In addition, specific RBPs are able to control the nuclear transport, translation efficiency and stability of mRNA through polyadenylation. Polyadenylation is a two-step process in which the transcript is first cleaved between the highly conserved AAUAAA sequence upstream and a degenerate U/GU rich sequence downstream of the cleavage site, after which the poly(A) polymerase adds the poly(A) tail to the cleavage product [35]. Apart from replication-dependent histone mRNAs, all eukaryotic mRNAs are edited to acquire a 3′ poly(A) tail of roughly 200 nucleotides. Following transcription, splicing and 3′end processing, RBPs contribute to the export of mRNA in a three-step process which results in the generation of a cargo-carrier complex in the nucleus, translocation of the complex through the nuclear pore complex, and the release of the cargo in the cytoplasm with subsequent recycling of the carrier. Following which RBPs orchestrate the localisation of transcripts to specific regions of the cells as well as translational regulation, including mRNA turnover, to control gene expression and stability. Moreover, studies have revealed that RBPs can bind to RNA through the chemical modifications in the 5′ cap, 3′ poly(A) tail and/or multiple internal marks and mediate RNA editing, the most prevalent type of RNA modification coordinating the conversion of adenosine to inosine and thus the alteration of RNA sequences through the insertion, deletion or substitution of nucleotides diversifying the transcriptome and thereby imposing a new layer of regulation for gene expression [25,36,37,38]. 

Unsurprisingly, proper functioning of these intricate post-transcriptional manipulations of RNA have shown to be essential for cell physiology and defects in either RBPs or RBP-regulated RNA network to be heavily associated with the onset and progression of pathological disorders. Due to the clear importance of RBPs in deciphering between health and disease states research to dissect the complex PTGR networks in various cellular systems is of great interest.

### Uncovering the Roles of RBPs

Despite their centrality in cell function, much is still largely unknown of RBPs evidenced by at least a third of known RBPs having either unknown or inferred biological functions. This was further emphasised in a recent study which investigated 1217 RBPs encoded by the human genome and found only 73% of the RBPs to have assigned RNA biological functions or a known structural RNA-binding motifs [39]. Consequently, to aid our knowledge and understanding of RBPs several in vitro experimental approaches to identify RBPs comprehensively in living cells have emerged including the use of immobilised RNA probes, RNA magnetic beads, or arrayed proteins. In summary, immobilised RNA probes are incubated with cell extracts and used as bait to identify RBPs using quantitative mass spectrometry. This method alone has had great success in uncovering numerous novel RBPS as well as uncover RBPs with distinct specificities across cell types [40,41]. Similarly, quantitative mass spectrometry has been used to identify RNA binding proteins following the incubation of purified polyadenylated cellular RNA immobilised on oligo(dT) magnetic beads with cell extracts [42]. In addition, arrayed proteins have also been used as bait to identify RBPs when incubated with fluorescently labelled RNA, allowing for the identification of RNA binding through measuring fluorescence intensity and protein spots, analogous to DNA microarrays [40,43,44].

Alternatively, another approach to uncover native protein–RNA interactions known as interactome capture (RIC) has emerged and led to both the identification and insight into the functions of hundreds of RBPs, further developing our understanding of RBP-RNA interactions. This method uses ultraviolet light irradiation to covalently link proteins to RNA positioned in direct proximity followed by incubation of denatured cell lysis with poly(A) RNA on oligo(dT) beads and the identification of all bound proteins by quantitative mass spectrometry [45]. Recently, click chemistry-assisted RNA interactome capture (CARIC), revealed 597 proteins to bind to both poly(A) and non-poly(A) RNAs, of which 130 RBPs had no prior RNA-binding annotation. This was significant as it not only uncovered novel RBPs but demonstrated for the first time that these newly discovered RBPs may possibly target both coding and non-coding RNAs, implying the involvement of non-coding RNAs in gene regulation network and biological processes too [46]. Also using ultraviolet light irradiation, enhanced crosslinking immunoprecipitation (eCLIP) allows for the identification of RBPs with single nucleotide resolution. In this approach, following irradiation, cell lysis and limited digestion to fragment RNA, protein-RNA complexes are immunoprecipitated with a specific RBP targeting antibody and resolved by denaturing gel electrophoresis. The RNA is subsequently converted to cDNA and the RBP bound regions isolated by high-throughput sequencing [47,48]. Moreover, numerous databases such as RBPDB [49], RBPmap [50], ATtRACT [51], hRBPome [52], oRNAment [53] have subsequently emerged to collect and store information about RBPs including their features and attributes which have further helped develop our understanding of RBP functions across various species. 

## 3. RBPs: Therapeutic Targets for Vascular Disease

As stated, both ECs and SMCs are integral to vascular health. The examination of living cardiac tissues and vessels is largely unattainable. Protocols to effectively derive iPSCs from patients as well as their subsequent differentiation into associated cell types to create effective disease models have therefore emerged; models that have since allowed for the elucidation of novel pathogenic mechanisms [54,55]. Consequently, iPSC derived disease models have repeatedly been used as a strategic tool to evaluate the involvement of specific cell types in vascular disorders. The suitability of iPSC derived cells as effective surrogates for their examination in disease states has been extensively evaluated. Sa et al previously evaluated and demonstrated that ECs differentiated from iPSCs compared to pulmonary arterial ECs derived from the same patients, with either heritable or idiopathic pulmonary arterial hypertension, share common functional abnormalities and gene expression patterns [56]. Since patient derived vascular cells have revealed novel disease pathogenic mechanisms [57,58,59,60]. iPSCs have also been utilised to assess known risk factors that contribute to vascular complications. Diabetes Mellitus (DM), for example, has repeatedly been associated with the onset of vascular dysfunction. iPSCs derived from diabetic individuals have helped identify defects and dysregulated networks involved in diabetic endotheliopathy aiding our understanding of the vascular complications associated with DM [61]. Moreover, a protocol to generate iPSC derived blood vessel organoids which recapitulated the morphological, functional, and molecular features of the human microvasculature recently emerged, allowing for in depth analysis of diabetic blood vessel characteristics [62,63]. These studies therefore validate the importance of stem cell advances in aiding vascular research through providing viable alternatives to primary cell types for modelling and identifying regulators of vascular health. 

Both ECs and SMCs are known to have their gene expression profile regulated by both transcriptional and post- transcriptional mechanisms [64,65,66]. As such, research has identified RBP responses in both ECs and SMCs, as well as other vascular cell types such as pericytes [26,67], cardiomyocytes [68] and inflammatory cells (specifically the differentiation of monocytes to macrophages) [69], to environmental cues, injuries, and risk all with the aim of limiting damage, maintaining homeostasis and restoring function. These reparative modifications, however, have also shown to trigger the onset, persistence, and aggravation of numerous diseases. This RBP controlled phenotypic switching can therefore determine between health and disease states. Due to the extensive involvement of RBPs in regulating vascular cell types, RBPs and phenotypic switching have been repeatedly linked to vascular disease onset and progression. Mechanisms behind these phenotypic switches in vascular cells have been extensively summarised in previous reviews [16,34]. Whilst we recently discussed the roles of RBPs within vascular function, dysfunction, and disease [70]. An overview of the roles of some RBPs within vascular health and disease can be found in Table 1. Here we therefore focus on the potential that controlling harmful responses of RBPs has as a beneficial therapeutic strategy to alleviate disease development, simultaneously stressing the importance that investigation into RBPs and RNA editing has on our understanding of vascular health evidenced here through the in-depth analysis of the RBP QKI family. 

**Figure 2:**
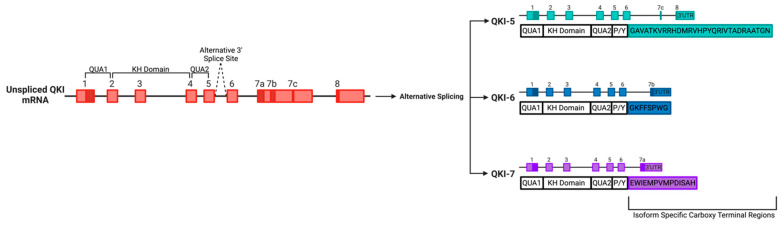
The Quaking (QKI) locus expresses alternatively spliced RNA-binding proteins belonging to the signal transduction and activation of RNA (STAR family).A schematic displaying the three major isoforms of QKI, namely, QKI-5, QKI-6 and QKI-7, yielded from the alternative splicing of the QKI gene, displayed with both exons and introns.

The QKI RBPs belong to the evolutionally conserved Signal Transduction and Activation of RNA (STAR) family. Analysis of the family has revealed QKI to contain a KH RNA-binding motif domain, N-terminal Qua1 and C-terminal Qua2 domains as well as multiple phosphorylation sites [87]. Due to alternative splicing multiple isoforms of QKI are yielded, all of which share RNA-binding domains and differ only in their carboxy-terminal ends [88]. In the last decade the three major QKI isoforms, namely QKI-5, QKI-6 and QKI7, have emerged to be critical regulators of the vascular system (Figure 2). Historically, the main function of QKI was believed to be in the involvement of the nervous system. In fact, QKI was named following the discovery that a mutation in the now referred to QKI gene resulted in tremors, “quaking”, due to the myelination defects in both the central and peripheral nervous system [89]. Analysis of the QKI isoforms, using both bioinformatics and in vitro techniques such as systematic evolution of ligands by exponential enrichment (SELEX) and database for annotation, visualization and integrated discoveries (DAVID), have since revealed the targets of QKI to most likely be involved in a range of activities including development, cell adhesion, organogenesis, transport and cell differentiation, morphogenesis, cell growth, maintenance and cell communication [90]. Since, QKI has proven to be essential for the appropriate function of a range of cell types including ECs and SMCs. 

**Figure 3:**
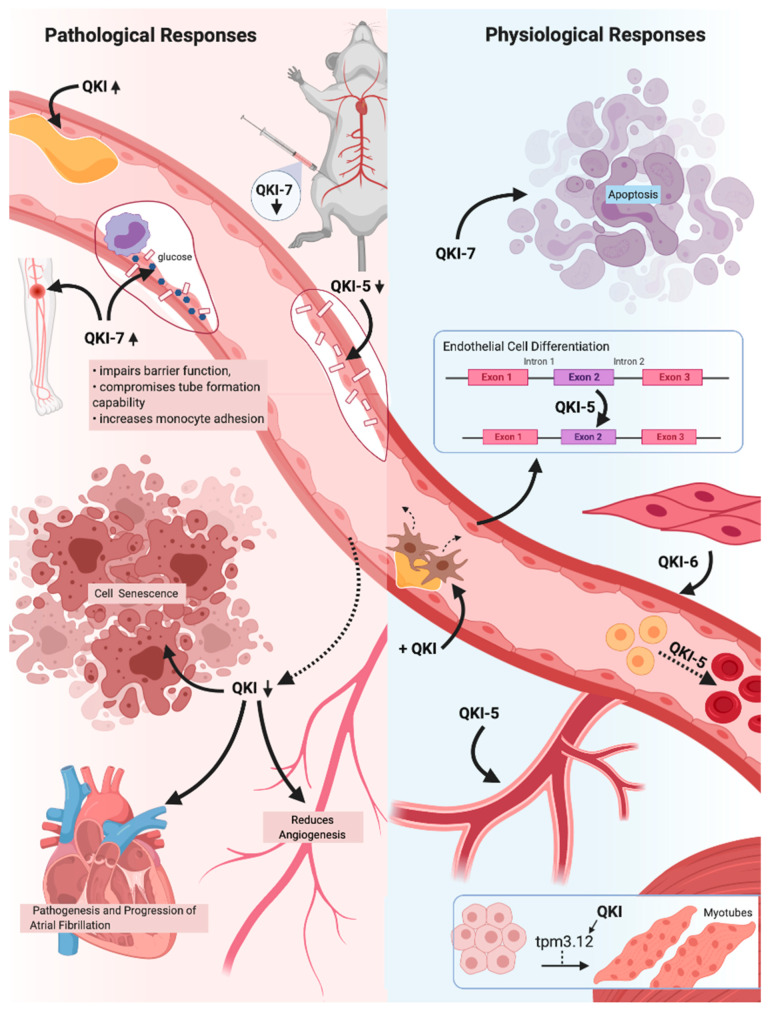
A schematic overview of the critical pathological and physiological responses the (cardio)vascular system exhibits in response to the three major Quaking (QKI) isoforms. QKI expression is known to drastically increase in response to vascular injury, suggesting reparative roles of the QKI isoforms. QKI reduction results in an inhibition of angiogenic and proliferative capabilities, as well as promotes cell senescence. Reduction in atrial myocytes specifically results in atrial fibrillation, whilst a lack of QKI-5 in endothelial cells (ECs) greatly impairs cell-to-cell adhesions and barrier function. QKI expression has also been shown to be vital in numerous cellular processes, including angiogenesis, erythropoiesis and EC, vascular smooth muscle cells (SMCs) and myotubule differentiation. In addition, QKI expression can ameliorate pathogenic fibroproliferative responses to vascular injury, whilst QKI-5 and QKI-6 expression is able to improve vascular cell qualities. Overexpression of QKI-7, however, has been shown to induce apoptosis and to drastically impair EC function, indicating a pathogenic role of QKI-7 expression within the (cardio)vascular system. The QKI isoforms therefore have great promise for use in vascular therapy due to their vital roles within (cardio)vascular health and disease states.

Whilst an initial role for QKI in vascular development was first suggested following Drosophila studies [91], it was not until the discovery that QKI null mice were unable to develop past embryonic day 10.5 that the importance of QKI to vascular health was confirmed. QKI knockdown in mice embryos resulted in defective vascular remodelling, immature endothelial tube structures and consequently the development of abnormal vitelline vessels [92,93]. Since the expression of the major QKI isoforms has been repeatedly found in both ECs and SMCs as well as during cardiac development [94]. Immunohistochemical and cell biological analyses have revealed the different isoforms to have distinct localisations and expression patterns; QKI-5 is mainly restricted to the nucleus, QKI-6 can be isolated in both the nucleus and cytoplasm whilst QKI-7 is predominantly found within the cytoplasm [95,96], suggesting the isoforms to have unique roles in cellular functions and the QKI carboxy-terminal to have an important role in RNA-processing functions. An overview of the key physiological and pathological roles of QKI in the vascular system can be seen in Figure 3.

Of the three major isoforms QKI-5 is the most abundantly expressed in ECs [64]. Using iPSC technologies, QKI-5 has been highlighted as a vital regulator of Signal transducer and activator of transcription 3 (STAT3) stabilisation and vascular endothelial growth factor receptor 2 (VEGFR2) activation during EC differentiation [79]. Moreover, due to its abundant expression in both macro- and micro-vascular ECs, it was also believed to be heavily involved in maintaining EC function. Subsequent exploration of iPS-ECs revealed QKI-5 to bind to VE-cadherin and β-catenin and mediate and increase their translation, both of which are widely accepted to have central roles in EC cell-cell adhesions and barrier function [79]. Moreover, in vivo and in vitro knockdown experiments severely attenuated endothelial barrier function, whilst induction of QKI-5 significantly improved and induced angiogenesis, blood flow recovery and neovascularisation [79]. Collectively, as expected due to the high expression profile, multiple studies have demonstrated the importance of QKI-5 for appropriate vascular function.

Unsurprisingly, QKI-5 downregulation has been associated with various vascular disease pathologies also. Analysis of diabetic hearts for instance found QKI-5 to be significantly downregulated and enhance ischemic intolerance through promoting Forkhead Box O1 (FOXO1) activation, mediating myocardial cell death and increasing the susceptibility of ischemic injury [97]. Introduction of QKI-5 inhibited ischemia by suppressing apoptosis as well as reducing nitrosative and endoplasmic reticulum stress via regulating FOXO1 degradation [97]. Research since has found QKI-5 expression to be linked to a novel hypoxia/reoxygenation induced cardiomyocyte apoptosis pathway and therefore injury, which is significantly suppressed upon QKI-5 expression [98]. Similarly, QKI has been found to be significantly downregulated in non-diabetic human failing hearts, displaying a crucial role of QKI in maintaining heart function [99]. Moreover QKI-5 downregulation was linked to heart failure associated with the chemotherapy drug doxorubicin. Treatment of doxorubicin resulted in QKI-5 knockdown in both human iPSC derived CMs and rodent CMs subsequently resulting in increased apoptosis and atrophy [99]. Whilst QKI-5 overexpression significantly attenuated heart failure through regulating the expression of specific circular RNAs derived from the from the Titin (Ttn ), Formin homology 2 domain containing 3 (Fhod3 ) and Striatin/calmodulin-binding protein 3 (Strn3 ) genes [99]. Similarly, the second major isoform, QKI-6, has also shown to have fundamental roles in determining between health and disease states. Not only has QKI-6 shown to be pivotal in vascular SMC differentiation [78,100], an upregulation of QKI-6 has been found in response to vascular injuries, indicating to a reparative role of QKI-6 in guiding vascular repair too [65]. Accordingly, studies have found the introduction of QKI-6 in disease states to be associated with the reversal of vascular dysfunction such as atrial fibrillation [101]. Due to the fundamental roles of QKI-5 and QKI-6 in vascular cell health, restoration of expression levels serves as a beneficial therapeutic strategy. Moreover, combined overexpression of both QKI-5 and QKI-6 has shown to result in vascular cell types with greater properties [78], further indicating their potential to be used in vascular therapy.

As stated, the differences between the isoforms resides within the C-terminal. Whilst the C-terminal of QKI-5 and QKI-6 predominantly regulate the localisation of their proteins, the unique 14 amino acids comprising the C-terminal of QKI-7 has shown to have key roles in increasing translation of mRNA by promoting the polyA tail extension of target mRNAs via the recruitment of poly(A) RNA polymerase GLD2 (PAPD4) [102]. Moreover, the C-terminal of QKI-7 can control cell survival by inducing apoptosis. The co-expression of both QKI-5 and QKI-6 alongside QKI-7 was found to promotes cell survival by orchestrating the nuclear translocation of QKI-7, preventing apoptosis [96]. Yet, if QKI-7 is more heavily expressed than the other isoforms, QKI-7 remained localised in the cytoplasm and induced cell death, indicating a necessity for the correct balance of the alternatively spliced QKI isoforms for both their normal function as well as cell viability. This also provides evidence that the C-terminal sequence of QKI-7 may function as a ‘life or death sensor’ monitoring the balance between the three. 

QKI-7 has also recently been identified to have a significant negative impact on cell function, contrary to the other QKI isoforms. Analysis of iPSC derived diabetic ECs revealed CUG-BP and hnRNPM regulation of QKI-7 to be abnormal, resulting in its overexpression [66]. Similarly previous research has also isolated QKI-7 to be the most abundantly expressed QKI isoform in ECs exposed to high shear stress [64], indicating a role for QKI-7 in vascular dysfunction. Analysis of QKI-7 overexpression in ECs has revealed QKI-7 to impair barrier function, compromise tube formation capability and increase monocyte adhesion, three hallmark features of endothelial dysfunction, through the regulation of critical EC related genes [66]. In particular, QKI-7 was found to bind and promote the degradation of vascular endothelial cadherin (CD144), Neuroligin 1 (NLGN1) and TNFα-stimulated gene-6 (TSG6). Deeper analysis also isolated QKI-7 overexpression to disturb the angiogenic capabilities of ECs. This research consequently implies a crucial role of QKI-7 in mediating the vascular complications associated with DM; an association which has been long established since the Framingham Study in 1979 and has since been repeatedly confirmed [103]. Importantly, targeting QKI-7 in vivo had the ability to restore EC function [66]. Restoration of QKI-7 function may therefore serve as a potential therapeutic strategy to alleviate diabetic related EC dysfunction and consequently slow the development of diabetic cardiovascular complications.

## 4. RBP-Based Therapeutic Strategies

**Figure 4:**
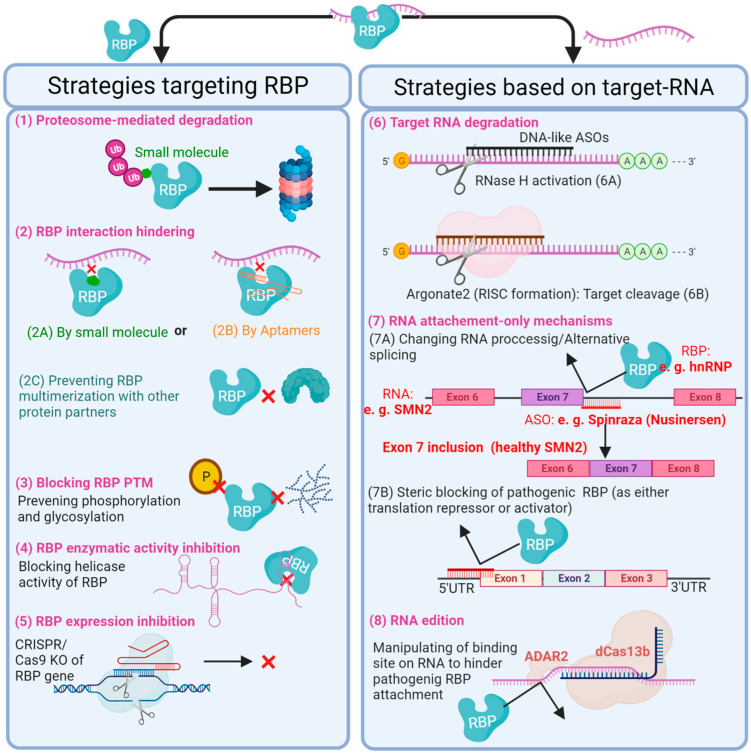
A figure displaying the current potential RNA-binding protein (RBP)-based therapeutic strategies to post-transcriptionally control cellular phenotype, with the aim of restoring or preventing vascular disease states. RBP-based therapeutic strategies either focus on inhibiting the action of the RBP itself or the target RNA. For example, small molecules and Aptamers can be designed to inhibit the action of RBPs either by tagging a specific RBP for degradation by proteasome (1) or by blocking the RBP interaction with either its target RNA or functional protein partner (2A–C). In addition, inactivation of RBPs can be achieved through the prevention of post-translational modifications (3). Another direct therapeutic approach is to block the enzymatic activity of a specific RBP (4) or to knock out its gene by CRISPR/Cas9 (5). RNA-targeting therapeutics, however, could be potentially used to reverse the effects of pathogenic RBPs on mRNA stability, alternative splicing and translation of target RNAs. RNA-based therapeutic tools, such as antisense oligonucleotides (ASOs), siRNAs, miRNAs and CRISPR/Cas, can be exploited to induce degradation of the target RNA independent from RBP (6A and B), block or manipulate interaction between an RBP and its RNA target through RNA occupation only (7) or RNA edition (8). For the latter, an RNA editor such as RNA adenosine deaminase 2 (ADAR2), with the help of the CRISPR/dCas13b system, can introduce point mutations in target RNA; it thereby has the potential to recapitulate a new RBP binding site, rescue a known pathogenic binding site or create a premature stop codon to spoil functionality of target RNA, for example. Within the figure, validated examples are shown in red text. RISC, RNA-induced silencing complex; KO, knockout; PTM, post-translational modifications; ASO, antisense oligonucleotide; SMN2, survival of motor neuron 2 gene.

As discussed, RBPs typically fall into one of two categories: pathogenic or protective. Based on this, efforts of designing RNA-based therapeutics should be focused on mimicking protective RBPs or inhibiting pathogenic RBPs to restore health and prevent disease-causing function, respectively. Every RBP has unique domains to bind its target RNAs in sequence-dependent fashion with structural conformation [30]. This provides therapeutic opportunity by developing drugs able to target specific RBPs or an RBP-RNA interaction (Figure 4) thereby limiting disease progression. To this end, attractive emerging potential RNA-based therapeutics include anti-sense oligonucleotide (ASO), aptamers, small interfering RNAs duplex (siRNAs), microRNAs, synthetic mRNAs as well as some CRISPR/Cas technologies (comprehensively reviewed by [104]). These therapeutic interventions can lead to the favourable knockdown/degradation or overexpression of specific target RNAs; alter the splicing by-product of pre-mRNA via masking the splice site or recruitment of splicing factors; prevent translation of mature mRNA by hindering its attachment to the ribosome; or modify the sequence of RNA by editing mutated RNA or inserting a mutation into the RNA to restore or prevent binding of a specific RBP, respectively [104,105].

RBPs have continually been found to be involved in regulating the transcriptome during phenotype switching in CVDs. In addition, studies have shown many RBPs to be unique to various cellular components of the cardiovascular system. For example, Liao et al. using mRNA interactome capture and RBDmap identified 1,148 RBPs, 393 of which were unique to cardiomyocytes and many of these RBPs linked to heart diseases [68]. Moreover, as heavily discussed in this review, research has repeatedly highlighted the significance of restoring RBPs to typical physiological function. As such understanding the full impact of RBPs within the cardiovascular system and their involvement in disease progression is vital for the reversal may have the potential to become effective therapeutic strategies. As was the case with the first FDA approved RNA-targeting therapeutic oligonucleotide, commercially named SpinrazaTM (Nusinersen, Biogen) [105,106] that was designed to target spinal muscular atrophy (SMA). SMA is a disease that arises from a splicing deficiency in the Survival Motor Neuron 2 (SMN2) gene. SpinrazaTM is an ASO with the ability to bind to SMN2 pre-mRNA and hinder the attachment of the heterogeneous nuclear ribonucleoprotein (hnRNP); thereby exposing the splice site responsible for inclusion of exon 7 to produce a full-length, functional SMN2 mature mRNA, which ameliorates SMA disease progression by restoring motor neuron function [107,108]. Consequently RNA-targeting therapeutics have great promise to be able to extend the domain of pharmaceutics to currently ‘undrugable targets’ beyond what has already been achieved by small molecules and biologics due to their centrality in the deregulation of post-transcriptional events. Both the advantages and challenges of RNA based therapies have been extensively reviewed previously by Lieberman [104]. Moreover, as stated, HuR expression is upregulated and correlated with poor prognosis in coronary artery disease [11]. HuR may therefore serve as a potential diagnostic and prognostic biomarker. The role of RBPs as potential diagnostic, prognostic and therapeutic biomarkers in vascular diseases is therefore another important research focus as early diagnosis is heavily associated with an improved prognosis. 

## 5. Conclusions

Despite only one RBP-based therapy currently being approved for use in patients, widescale clinical applications of RBPs are inevitable. Recent developments have highlighted a pivotal role for RBPs and RNA interactions in mediating between health and disease states. Therefore, the ability of RBPs to control cellular phenotypes has great potential in therapeutics through restoring or preventing disease phenotypes. Due to the large uncertainties surrounding RBP functions, it is essential we deepen our understanding of RBPs to unveil their full potential and unlock new therapeutic avenues for currently undruggable targets, as seen following the comprehensive studies of the QKI isoforms. Considering recent developments, it is also important to consider the therapeutic potential of RBPs with respect to both treatment and generation of effective models within the cardiovascular system. With this in mind, future work regarding disease pathogeneses should evaluate specific roles of RBPs within both health and disease states in order to reveal novel routes of pathogenesis and treatment strategies, the groundwork for which has already been laid through the development of databases such as RBPDB, RBPmap, AttRACT, hRBPome and oRNAment.

## Figures and Tables

**Table 1:** Roles of RBPs within Vascular Health and Disease.

RBPs	Clinical Outcome	References
ANKRD17	ANKRD17 deficient mice are embryonic lethal due to cardiovascular defects as a result of incomplete vascular maturation.	[71]
HuR	HuR has a range of roles across the vasculature. For example, HuR has critical roles in vascular smooth muscle contraction and maintains blood pressure regulation. In addition, HuR regulates multiple angiogenic factors and promotes cell proliferation and migration of endothelial cells. Deficiency has been associated with decreased angiogenesis as well as abnormal vasculature. HuR has also arisen as a viable therapeutic target for pathological cardiac hypertrophy and heart failure and expression to correlate with poor prognosis in coronary artery disease.	[19,72,73,74,75,76,77]
QKI	A lack of QKI-5 in endothelial cells impairs cell-to-cell adhesions and barrier function and is associated with multiple vascular diseases, including ischemia. QKI-5 and QKI-6 expression improves vascular cell health as well as encourages neovascularisation and blood flow recovery in a hind limb ischemia model. QKI-7 upregulation disrupts endothelial cell–cell barrier, compromises angiogenesis and enhances monocyte adhesion.	[64,66,69,78,79]
SHARPIN	A role for SHARPIN has emerged in blood vessel health as well as angiogenesis. Moreover, SHARPIN has been shown to have differential and context-dependent roles in platelets to regulate inflammatory and integrin adhesive functions.	[80,81]
ZFP36/TTP	ZFP36 expression can suppress atherosclerosis. Deficiency reduces blood pressure, triggers vascular inflammation, reduces relaxation upon acetylcholine and orchestrates endothelial cell dysfunction.	[82,83,84,85,86]
